# Deflection of Steel Fiber Reinforced Concrete Beams Based on Waste Sand

**DOI:** 10.3390/ma13020392

**Published:** 2020-01-15

**Authors:** Jacek Domski, Mateusz Zakrzewski

**Affiliations:** Faculty of Civil Engineering, Environmental and Geodetic Sciences, Koszalin University of Technology, 75-453 Koszalin, Poland; mateusz.zakrzewski@tu.koszalin.pl

**Keywords:** SFRC—steel fiber reinforced concrete, waste sand, deflection, beams

## Abstract

The article describes the selected methods of calculating the deflection of steel fiber reinforced concrete beams. Additionally, the results of the study on the deflection of steel fiber reinforced concrete beams based on waste sand are presented. This paper compares deflections measured during the four point bending test of the steel fiber reinforced, waste sand fine aggregate concrete beam with values determined in accordance with Eurocode 2, the proposal of Tan, Paramasivam, and Tan, the modified method of Alsayed, Bywalski, and Kaminski, and Amin, Foster, and Kaufmann’s method. The analysis conducted shows that the best accordance with the study and calculation results was obtained by using the modified Alsayed method.

## 1. Introduction

The main relationship defined in bend tested steel fiber reinforced concrete beams is the relation between the load and deflection [1]. Based on the above relationship, the toughness, toughness factor, and flexural toughness indexes can be determined for small elements, e.g., 100 mm × 100 mm × 300 mm. The value of the deflection at which the toughness factor in bending is determined is 1/150 of the tested beam span. However, to define the flexural toughness indexes in bending, e.g., I30 and I100, the deflection value at cracking should be multiplied by 15.5 and 50.5, respectively. There are many proposals for the definition of the above mentioned parameters of bent small beams [2,3,4,5,6], but they do not apply to the description of the deflection of bend tested steel fiber reinforced concrete beams with bar reinforcement, because the steel bars considerably influence the load–deflection curve’s character [7].

The methods used for the calculation of steel fiber reinforced concrete element deflections initially comprised small beams without bar reinforcement [4,8]. Among the first researchers who, in 1987, analyzed both bar reinforcement and steel fibers were Craig [9], Liqiu and Guofan, and Lim, Paramasivam, and Lee [10]. In 1992, Hsu, He, and Ezeldin [11] proposed a method for the deflection determination of single span beams. The next two calculation proposals based on the modification of beam rigidity were developed in 1993. The first was proposed by Alsayed [12], whereas the second, which pertained to beams made of high strength concrete, was proposed by Ashour and Wafa [13]. Tan, Paramasivam, and Tan [14] proposed, in 1994, a modification of the Branson method (for plain concrete), which considered the impact of steel fibers on beam rigidity. In 1995, Ezeldin and Shiah [15] presented a method for the calculation of steel fiber reinforced concrete beam deflection both under temporary and long term load conditions. The next method was proposed by Bywalski and Kaminski in 2011 [16]. The method was based on Polish Code PN-B-03264 and took into account the relations between steel fibers and the moment of inertia for cracked and non-cracked concrete. In 2016, Amin, Foster and Kaufmann [17] presented a method based on the tension chord model. The modifications of the Kenel method contained the influence of the fiber on the second moment of the area of the cracked beam and the tension stiffening effect.

## 2. Selected Methods of Beam Element Deflection Calculation

To make the comparison of methods easier, all symbols were unified.

Notation:*A*_*s*1_Area of tensile longitudinal steel reinforcement;*A*_*s*2_Area of compressive longitudinal steel reinforcement;*b*Width of member;*d_f_*Diameter of fiber;*d*Distance from the center of gravity of the stretched reinforcement to the most compressed beam edge;*d*′Distance from the center of gravity of the compressed reinforcement to the most compressed beam edge;*E_c_*Modulus of elasticity of plain concrete;*E_cf_*Modulus of elasticity of steel fiber reinforced concrete;*E_s_*Modulus of elasticity of steel reinforcement;*F*Applied load;*f_ct_*Tensile strength of concrete matrix;*f*_0.5_Residual tensile strength calculated at a Crack Mouth Opening Displacement, CMOD = 0.5 mm;*h*Height of member;*I_cr_*Transformed moment of inertia for the cracked cross-section;*I_g_*Gross moment of inertia;*k_t_*Orientation factor;*l_f_*Fiber length;*M_a_*Applied moment;*M_cr_*Cracking moment;*n*Proportion of longitudinal steel reinforcement modulus of elasticity (*E_s_*) and steel fiber reinforced concrete (*E_cf_*);*n_f_*Proportion of steel fiber reinforcement modulus of elasticity (*E_f_*) and steel fiber reinforced concrete (*E_cf_*);*V_f_*Volume of steel fiber content in the entire mixture;*x*Compressed zone height.

Tan, Paramasivam, and Tan [14] proposed, in 1994, a method of deflection calculation based partly on the Branson method (1977) for plain concrete used in the American Concrete Institute (ACI) Building Code and applicable until today [18]. The main parameter describing the serviceability limit state here is the cracking moment *M_cr_*. In the method, the authors made an assumption that the impact of fibers on the moment of inertia could be omitted; therefore, the equation takes the following form:

(1) $$M_{\mathrm{cr}}=\frac{\phi_{\mathrm{cf}}I_{g}}{y_{t}}$$

where:

*y_t_*, distance from the center of gravity to the stretched cross-section edge;

*ϕ_cf_*, strength for the first crack in steel fiber reinforced concrete small beams; it can be defined using an empirical formula proposed in 1974 by Swamy and Mangat [19] (recommended by American Concrete Institute [20]), based on the so-called law of mixtures:

(2) $$\phi_{\mathrm{cf}}=0.843f_{r}\left(1-V_{f}\right)+2.93V_{f}\frac{l_{f}}{d_{f}}$$

where:

*f_r_*, concrete small beams’ flexural strength (N/mm^2^); it can be calculated as a product of constant 0.622 and the square root of the compression strength defined for cylinders (N/mm^2^).

To take into account the change of rigidity along the element length and the effect of tension stiffening, gross, transformed, and effective moments of inertia were used:

(3) $$I_{e}={\left(\frac{M_{cr}}{M_{a}}\right)}^{3}I_{g}+\left[1-{\left(\frac{M_{cr}}{M_{a}}\right)}^{3}\right]I_{cr}\leq I_{g}$$

where:

*I_cr_*, transformed moment of inertia for the cracked cross-section taken from the following equation:

(4) $$I_{cr}=\frac{bx^{3}}{3}+nA_{s1}{\left(d-x\right)}^{2}+\left(n-1\right)A_{s2}{\left(x-d^{\prime }\right)}^{2}+n_{f}A_{f}\frac{{\left(h-x\right)}^{2}}{3}+\left(n-1\right)A_{f}^{\prime }\frac{x^{2}}{3}$$

where:

*A_f_* and *A_f_′*, steel fibers in the compressed and stretched zone surface areas; the method of calculation was proposed in 1978 by Hannant, making them dependent on fiber effective length (η_1_) and their orientation before cracking (*η*_0_) and after (*η*_0_′):

(5) $$\begin{array}{l} A_{f}=\eta_{1}\eta_{0}^{\prime }V_{f}b\left(h-x\right)\\ A_{f}^{\prime }=\eta_{1}\eta_{0}V_{f}bx. \end{array}$$

The values of *η*_1_, *η*_0_, and *η*_0_′, applied in Equation (5) can be determined using the proposal presented in 1987 by Lim, Paramasivam, and Lee [10]. The effective length coefficient (*η*_1_) depends on the length of fibers used (*l_f_*) in relation to the critical value (*l_c_*); for *l_f_* < *l_c_*, *η*_1_ = 0.5, otherwise, it should be calculated using the following equation:

(6) $$\eta_{l}=1-\frac{l_{c}}{2l_{f}}$$

where:

*l_c_*, critical fiber length; it can be taken from a formula developed in 1974 by Swamy and Mangata in 1974 [19]:

(7) $$l_{c}=\sigma_{fu}\frac{d_{f}}{2\tau }$$

where:

*σ_fu_*, fiber ultimate tensile strength;

*τ*, average interfacial bond stress between the fiber and matrix.

The fiber orientation factors before (*η*_0_) and after cracking (*η*_0_*′*) depend on the fiber length (*l_f_*) and element cross-section dimensions (*b* × *h*). Those values can be taken from the following equations:

(8) $$\eta_{0}=\frac{\int_{0}^{\bar{\rho }}\int_{0}^{\bar{\theta }}\cos^{4}\theta \;\cos^{4}\rho \;d\theta \;d\rho }{\int_{0}^{\bar{\rho }}\int_{0}^{\bar{\theta }}d\theta \;d\rho }$$

(9) $$\eta_{0}^{\prime }=\frac{\int_{0}^{\bar{\rho }}\int_{0}^{\bar{\theta }}\cos\theta \;\cos\rho \;d\theta \;d\rho }{\int_{0}^{\bar{\rho }}\int_{0}^{\bar{\theta }}d\theta \;d\rho }$$

where: 

$\bar{\theta }=\sin^{-1}\left(h/l_{f}\right)\leq \pi /2$ and $\bar{\rho }=\sin^{-1}\left(b/l_{f}\right)\leq \pi /2$.

Based on Equations (4) to (9), the effective moment of Equation (3) is determined for fixing the effective rigidity under the assumption that the steel fiber reinforced concrete modulus of elasticity (*E_cf_*) can be taken from the following equation:

(10) $$E_{\mathrm{cf}}=\left(1-\eta_{1}\eta_{0}V_{f}\right)E_{c}+\eta_{1}\eta_{0}V_{f}E_{f}$$

where in the modulus of elasticity for plain concrete (*E_c_*) is calculated as a product of 4.73 and the square root of the compression strength defined for cylinders.

The final steel fiber reinforced concrete beam deflection is determined through the adoption of *E_cf_*, *I_e_* as the element’s effective rigidity.

In 1993, Alsayed put forward a proposal for the calculation of steel bar and fiber reinforced concrete beam deflection [12]. It was based on the determination of a steel fiber reinforced concrete beam’s rigidity *(EI)_ef_*, taking into account the influence of steel fibers through multiplication of the gross moment of inertia (*I_g_*) by the K coefficient. According to the method of the author, the influence of steel fibers on the deflection reduction is perceptible only after element cracking, so the influence of fibers on the modulus of elasticity and on the effective moment of inertia was not taken into account here. Therefore, Alsayed’s considerations pertained to a situation when the bending moment is higher than the cracking moment (*M* > *M_cr_*). Therefore, the steel fiber reinforced concrete beam rigidity formula takes the following equation:

(11) $${\left(EI\right)}_{ef}=E_{c}\left(I_{e}+K\cdot I_{g}\right)$$

where:

*K*, coefficient taking into account the percentage of fibers and their slenderness ratio defined by the following equation:

(12) $$K=\alpha_{1}{\left(V_{pf}\frac{l_{f}}{d_{f}}\right)}^{\alpha_{2}}{\left(\frac{M_{cr}}{M}\right)}^{\alpha_{3}}\;$$

where:

*V_pf_*, percentage of steel fibers,

*α*_1_, *α*_2_, *α*_3_, coefficients defined by Alsayed based on his own research work, verified by the results of, among others, Swamy and Al-Ta’an [21]; *α*_1_ = 0.45; *α*_2_ = 2.0, *α*_3_ = 1.25.

The methods of calculation of the deflection limit state in beams with added steel fibers presented in the Introduction and described hereabove pertain to steel fiber reinforced concrete elements made of plain concrete. Considering the specific properties of the steel fiber fine aggregate concrete mixtures made with waste sand, the above presented Alsayed method was modified. The proposed change refers to Equation (11), where the steel fiber fine aggregate concrete modulus of elasticity should be used instead of the modulus of elasticity for plain concrete (*E_c_*). Such a course of reasoning was dictated by the fact that Alsayed considered the influence of added fibers only on the moment of inertia value, whereas it is a well-known fact that the fibers usually cause a change of the modulus of elasticity. Furthermore, Alsayed limited his considerations only to the cracked element analysis, and this does not cover the entire problem. Therefore, the first stage of deflection (for non-cracked elements) should be determined, consequently with the relation to the second stage calculations (for cracked elements), in accordance with the formulae given in the ACI Building Code 318 [18], taking into account the mechanical features of the steel fiber sand concrete.

In 2011, a method for deflection determination of steel fiber reinforced concrete was presented in [16]. The method included the growth of the moment of inertia caused by steel fibers’ content. The estimation of deflection used basic, well known methods. The means of neutral axis and moment of inertia determination included steel fiber distribution in the element. Steel fiber localization was approximated by a uniform distribution to simplify calculations. Substitute cross-sections for steel reinforcement (both steel bars and fiber) were calculated and included in the equations used for neutral axis *z_cr_* and moment of inertia determination. In the case of cracked concrete, only fibers from the concrete cross-sectional area subjected to a strain smaller than 2.5‰ were included in the calculations. The most general system of equations, which includes strains greater than 2.5‰, takes the form:

(13) $$\begin{array}{c} \left(\frac{b}{2}+\frac{\beta_{3}\alpha_{e,eff}^{fib}A_{s}^{fib}}{2h}\right)z_{cr}^{2}+\left(\alpha_{e,eff}A_{s1}+\alpha_{e,eff}A_{s2}\right)z_{cr}-\\ -\alpha_{e,eff}A_{s1}d-\alpha_{e,eff}A_{s2}d^{\prime }-\frac{\beta_{3}\alpha_{e,eff}^{fib}A_{s}^{fib}0.0025^{2}E_{c,eff}^{2}}{2hM_{a}^{2}}I_{cr}^{2}=0 \end{array}$$

(14) $$\begin{array}{c} I_{cr}=\frac{b}{3}z_{cr}^{3}+\alpha_{e,eff}A_{s2}{\left(z_{cr}-d^{\prime }\right)}^{2}+\alpha_{e,eff}A_{s1}{\left(d-z_{cr}\right)}^{2}+\frac{\beta_{3}\alpha_{e,eff}^{fib}A_{s}^{fib}}{4h}z_{cr}^{3}+\\ +\beta_{3}\alpha_{e,eff}^{fib}A_{s}^{fib}\frac{0.0025^{3}E_{c,eff}^{3}}{4hM_{a}^{3}}I_{cr}^{3} \end{array}$$

where:

*β*_3_, factor reducing the efficiency of steel fiber anchorage;

*E_c,eff_*, effective modulus of the steel fiber reinforced concrete including creep, calculated as in EN 1992-1-1 [22];

*α_e,eff_*, *α_e,eff_^fib^*, the proportion of the longitudinal steel reinforcement/steel fiber modulus of elasticity and effective modulus of steel fiber reinforced concrete.

The results are narrowed by additional geometrical constraints:

(15) $$0\leq z_{cr}\leq h$$

(16) $$0\leq I_{cr}\leq I_{g}$$

After those calculations, beam stiffness should be determined in accordance with the EN 1992-1-1 [22] standard.

**Figure 1:**
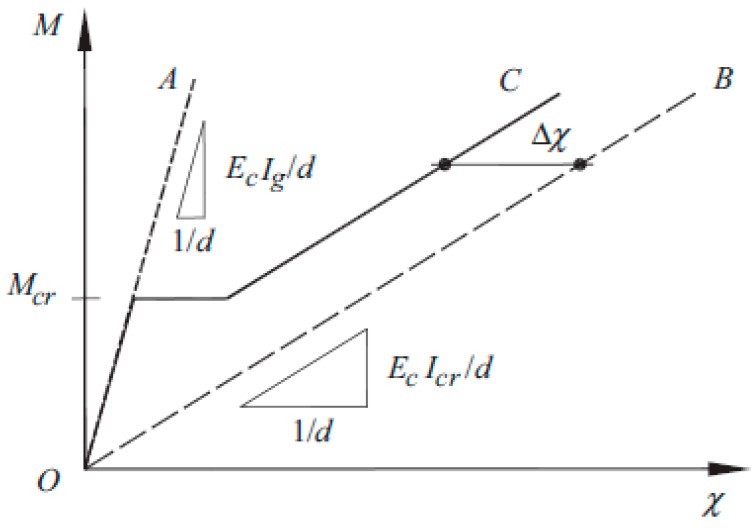
Moment–curvature relationship for RC members [23].

The method presented by Amin, Foster, and Kaufmann [17] was based on the tension chord model (Figure 1). This model was presented by Kenel [23] and others for unidirectional bend elements.

The modification contains the influence of fiber reinforcement on the moment of inertia for the cracked cross-section (*I_cr_*) and tension stiffening effect (distance between Lines C and B in Figure 1). The moment of inertia of a rectangular SFRC section can be taken as:

(17) $$I_{cr}=\frac{bx^{3}}{3}+nA_{1}{\left(h-x\right)}^{2}+\left(n-1\right)A_{s2}{\left(x-d_{sc}\right)}^{2}+nA_{F}\times \frac{{\left(h-x\right)}^{2}}{3}\;$$

The last term in Equation (17) accounts for the influence of the steel fibers in the cracked portion of the cross-section. *A_F_*, the cumulative area of fibers in the cracked portion of the cross-section, can be evaluated as:

(18) $$A_{F}=0.82\frac{V_{f}}{2k_{t}}\left(h-x\right)b$$

where *k_t_* is an orientation factor that can reasonably be approximated as:

(19) $$k_{t}=\frac{1}{0.94+\frac{0.6l_{f}}{b}}$$

**Figure 2:**
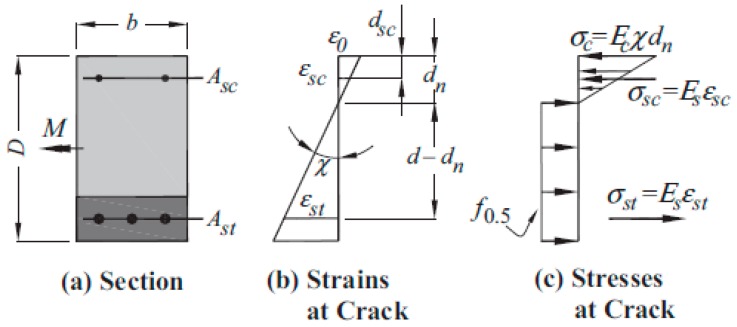
Strains and stresses on a cracked SFRC rectangular section in bending [17].

The compressed zone height *x* in Amin, Foster, and Kaufmann’s method can be determined by considering the force equilibrium of the cracked section and strain compatibility (Figure 2). Unlike the determination of *x* for plain RC members, an iterative procedure is required for SFRC as the assumed stress carried by the fibers *f*_0.5_ is independent of the induced strain. Amin, Foster, and Kaufmann did not describe what *f*_0.5_ precisely was. Most likely, it was the residual tensile strength calculated at a *CMOD* = 0.5 mm.

An important aspect of Amin, Foster, and Kaufmann’s proposal was the constant curvature offset Δ*χ* due to tension stiffening in RC members. The constant curvature offset Δ*χ* can be taken from:

(20) $$\Delta \chi =\frac{\left(0.75+\frac{1.25f_{0.5}}{f_{ct}}\right)bf_{ct}}{6A_{s1}E_{s}}$$

## 3. Composition of Concrete Mixtures and Test Elements’ Research Program

The sand and concrete matrix recipe was fixed based on an experimentally established relationship between the real water and pore volumes in the concrete mixture and the sand concrete properties. Then, the fixed sand and concrete mixture composition was modified by the addition of a superplasticizer and steel fibers. The water content was controlled to obtain a plastic consistency mixture [24]. The commercial concrete composition was developed by its Polish producer (Dźwigbet–Koszalin), for a fixed cube compressive strength of 45 MPa. The final compositions of the steel fiber sand concrete mixtures and the commercial concrete recipe are indicated in Table 1 and Table 2.

The deflection caused by a temporary load was tested on beams of dimensions 150 mm × 200 mm × 3300 mm. The test elements were made in 10 series that varied in type of concrete mixture (steel fiber sand concrete and plain commercial concrete), steel fibers applied (50/0.8 mm and 30/0.55 mm), the beam element longitudinal reinforcement ratio (0.6%; 0.9%; 1.3%; 1.8%), and application of compressed reinforcement. Each beam had vertical stirrups spaced every 130 mm. Each series comprised 2 beams, 6 cylinders, and 12 cubic samples (Table 3). Test elements were demolded after 24 h as of concrete pouring. Thermal and humidity conditions during concrete casting and hardening were uniform.

The longitudinal beam reinforcement was made of ribbed steel (34GS grade) diameter 8, 10, 12, and 14 mm, whereas the transversal reinforcement was made of smooth steel (St3SX-b grade) diameter 4.5 mm. Only ribbed steel was tested for its mechanical properties. The tests performed were aimed at the definition of the yield point, tensile strength, and modulus of elasticity of the reinforcement bars (Table 4).

## 4. Beam Element Test Methodology

**Figure 3:**
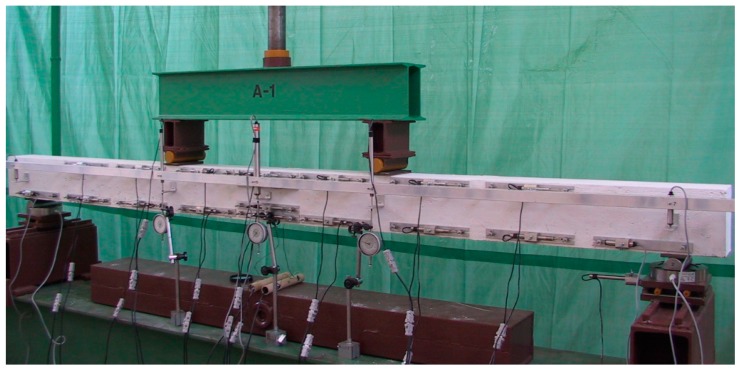
General view of the test rig for A-1 beam testing (Series A).

The rig for short term testing of beam elements was composed of a steel frame, hydraulic cylinder (fitted with the possibility to slide towards the top frame belt), and load control system. The static diagram of the tested elements was a single span freely supported beam loaded with two concentrated forces applied at 1/3 span between the support axes (Figure 3). The forces were applied via steel beam loaded with the hydraulic cylinder of 246 kN in range. On the bottom beam of the frame, two supports were mounted. Roller and pinned supports were designed in such way so that reaction measurement with tensometric force gauges could be performed (range, 100 kN). The load applied was controlled by reading support reaction force with the application of the SAD 256 computer data acquisition system (APIG Sp. z o.o., Łódź, Poland).

**Figure 4:**
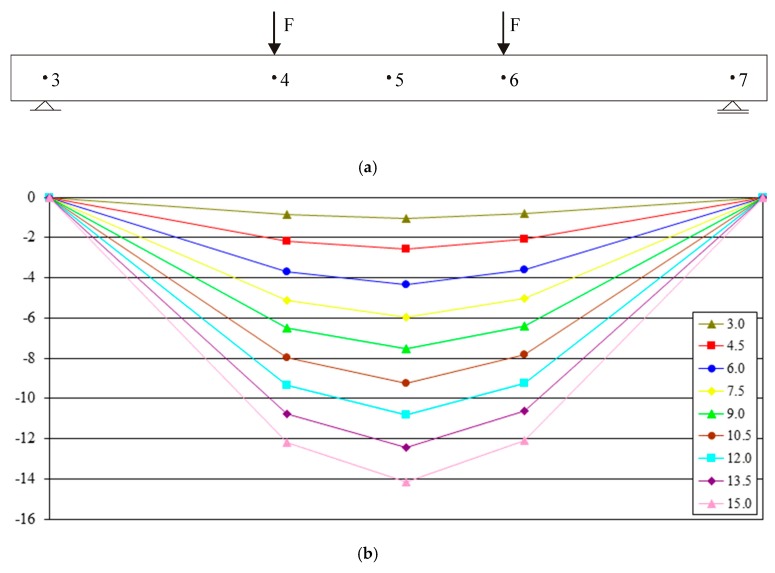
(**a**) beam loading scheme and distribution of measuring points; (**b**) B-1 beam deflection (mm) at particular load stages.

The system was also used for measurement of beam vertical displacements [27]. The deflection was measured with displacement sensors fitted in an aluminum strip fixed independently of the beam to the test the stand load bearing frame (Figure 3). The beam vertical displacement measurements were performed in five locations indicated by the angle bars fitted to the beam at half of its height, i.e.,:

- Mid-span (Figure 4, Item 5) via a sensor with a 100 mm range;
- Under load applying rollers (Figure 4, Points 4 and 6) via sensors with a 50 mm range;
- At support axes (Figure 4, Points 3 and 7) via sensors with a 10 mm range (Figure 4a).

## 5. Test Results and Analysis

The measurements of the displacements of selected beam characteristic points were performed in a dozen or so stages of loading (determined by the support reaction reading). The number of stages was selected on the basis of calculated beam load bearing capacity and conditioned upon their longitudinal reinforcement ratio. Displacement values were measured in each of the analyzed loading stages. Deflection was calculated in reference to supports’ vertical movement. Examples of B-1 beam graphs are given in Figure 4. The deflections obtained for each of the tested beams at the force application points (Points 4 and 6) differed insignificantly at all load stages. The maximum mean percentage error obtained for all analyzed beams was 5.8% (for the J-1 beam). These were small differences, which may have originated from small measurable values, particularly at the preliminary load stages. The differences for deflections in mid-span of beams of the same series, in particular load stages, gave an average error not exceeding 15% (with the exception of Series I beams). The average deflection value obtained for two beams of each series was used here for further analysis purposes.

**Figure 5:**
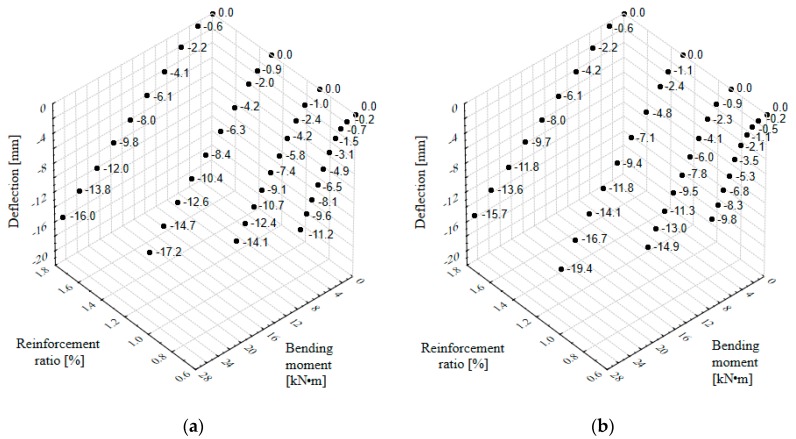
Relationship between the reinforcement ratio, bending moment, and deflection of fine aggregate and concrete beams with fibers: (**a**) 50/0.8 mm; (**b**) 30/0.55 mm.

Figure 5 shows the change of deflection (in beam mid-span) as a function of the maximum bending moment for various ratios of stretched reinforcement and two types of steel fibers applied. It appeared from the presented relationships that for the same load, the higher the stretched beam reinforcement ratio was, the smaller the deflections were. Therefore, in the case of steel fiber fine aggregate concrete beams, just like in the case of plain concrete beams, the rigidity depended on the total cross-section of steel reinforcement applied.

**Figure 6:**
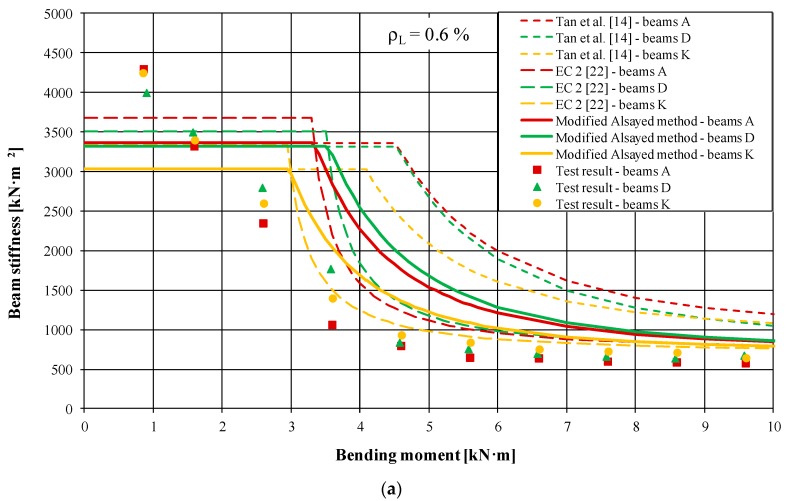

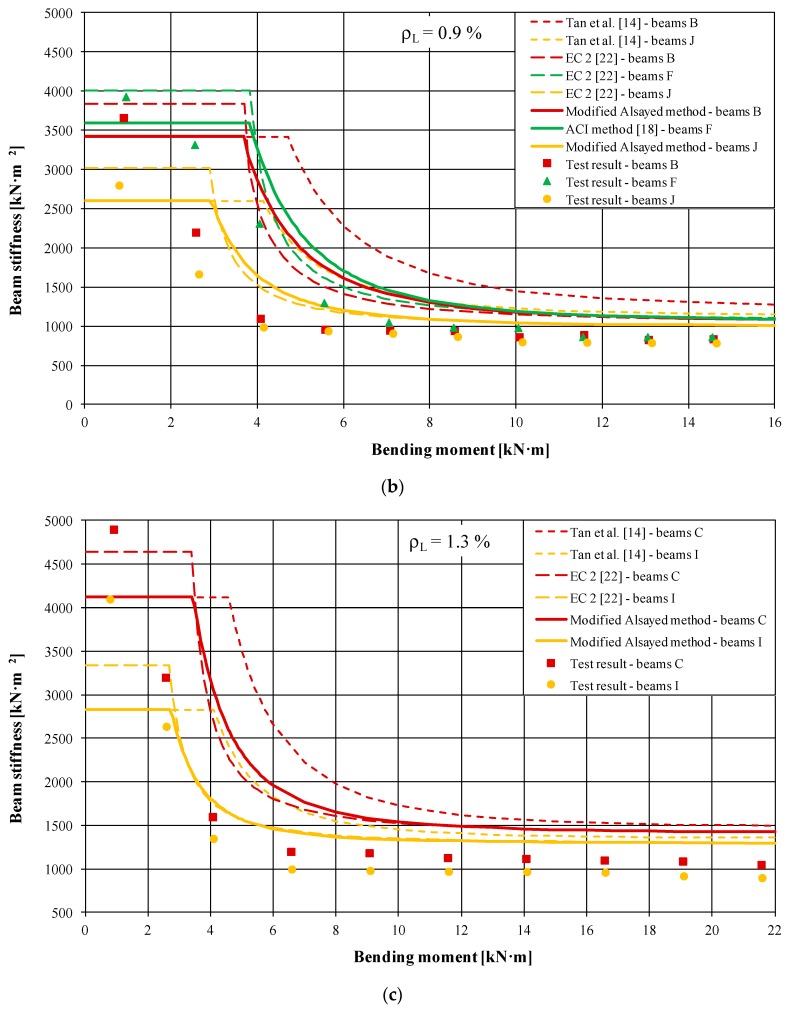

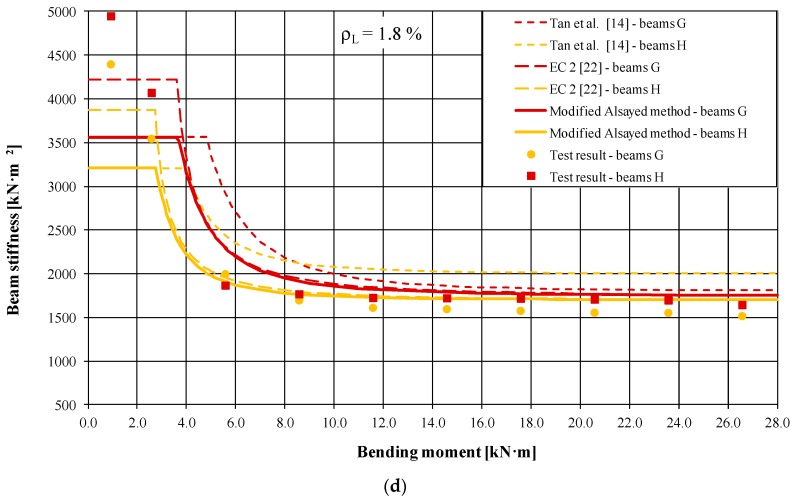
Relationship between rigidity and bending moment for beams of different stretched reinforcement ratios: (**a**) *ρ_L_* = 0.6%; (**b**) *ρ_L_* = 0.9%; (**c**) *ρ_L_* = 1.3%; (**d**) *ρ_L_* = 1.8%.

The influence of steel fibers and the ratio of reinforcement on beam rigidity was determined experimentally based on the product of the bending moment and the distance between the deformation measurement locations divided by the sum of deformation increments (compressing for concrete and stretching for steel reinforcement). Figure 6 comprises four graphs for various beam stretched reinforcement ratios (*ρ_L_*). The already mentioned Tan et al. method [14] and the proposal included in the Eurocode 2 [22], as well as slightly modified Alsayed rigidity definition method were applied here. The theoretical values were determined for each of the beam series (Figure 6, solid and broken lines) and put together with the results calculated from our own studies (points in Figure 6). It appeared from the analysis performed that beam rigidity decreased with increasing load and increased with the increasing of the stretched reinforcement ratio (*ρ_L_*). However, no significant impact of the applied steel fibers on the rigidity value was ascertained.

When comparing the graphs containing values calculated in accordance with the Eurocode 2 [22] proposal, the modified Alsayed and Tan et al. [14] method, it should be remembered that various rigidity values at the first stage were caused by different moments of inertia, *I_I_* and *I_g_*, respectively. Differences between the theoretical rigidity of cracked cross-section values decreased with the increase of the stretched reinforcement ratio. Discrepancies between the theoretical and experimentally acquired *E_c_·I* products occurred also in particular load stages. They were considerable at the first stage (before cracking), whereas in the second stage (after cracking), they decayed with the increase of the load and stretched reinforcement ratio. They could also be caused by incorrect assessment of the theoretical cracking moments and omission of the additional curvature caused by steel fiber fine aggregate concrete shrinkage. Numerous works on the deflection of beams made of plain concrete confirmed that the method of calculation in [22] provided, in general, better compatibility with the experimental results for elements featuring a higher reinforcement ratio. From the test results illustrated in Figure 6, it appeared that in steel fiber fine aggregate concrete elements, the change of rigidity and the influence of the load were an exponential function both for cracked and non-cracked cross-sections. This confirmed the correctness of the assumption about the continual rigidity of bent elements [16].

**Figure 7:**
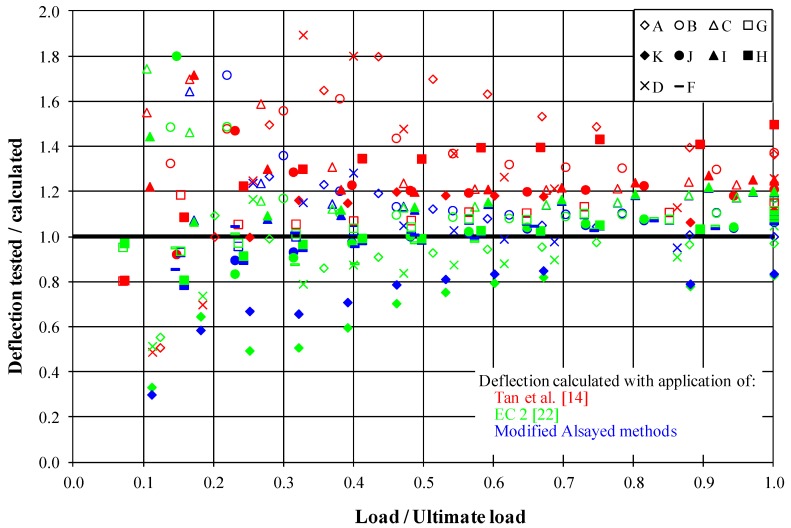
Analysis of the measured and calculated deflection of beams in their mid-span.

Figure 7 illustrates a comparison of the measured and calculated values of maximum deflections in subsequent load stages for all tested beams. The Tan et al. [14] and EC 2 [22] methods, as well as the authors’ modification of the Alsayed calculation method were used consistently in the calculations. It appears from Figure 5 that deflection values determined through the application of the Tan et al. [14] method were considerably lower than the measured values. This was probably caused by the fact that the proposed deflection determination method pertained to the steel fiber reinforced concrete beams and not the steel fiber fine aggregate concrete members. The Eurocode 2 method (for plain concrete) [22] gave deflection values that differed insignificantly from the test results. A similar situation pertained to the modified Alsayed method, for which the results were also close to the measured ones. A more accurate analysis of the presented results was accomplished for the operational loads, which are most important for civil engineering practice (Table 5).

The lowest deflection (10.12 mm) of the tested beams with the same ratio of reinforcement (B, F, and J beams) was obtained for the plain concrete, for which the highest rigidity was obtained (Figure 6). Similar, although a bit higher deflection (10.70 mm) was observed in the case of beams with longer fibers. It was ascertained, on the 50/0.8 mm beam containing fiber example, that the absence of reinforcement in the compressed element (D series) zone caused an increase of the deflection values in relation to the compressed reinforcement beams (A series), from 8.05 mm to 8.74 mm. It appears from Table 5 that for the beams of a low stretched reinforcement ratio (A, D, and K, 0.6%), the Eurocode 2 [22] method gave deflection values overestimated even by 25.8%. The values of the measured deflections in the remaining beams were higher than the calculated values by a max of 13.5%. This confirmed the earlier observation that the method in [22] allowed obtaining generally better compatibility with the test results for elements of a high reinforcement ratio. Deflection calculated by Amin, Foster, and Kaufmann [17] was lower than the measured value. The method of Bywalski and Kaminski [16] gave results close to those measured, except for D and K series, which had a lower longitudinal reinforcement ratio. The deflection values determined as per the Tan et al. [14] proposal were underestimated by a max of 38.8%. The mean error in this case was 21.0%, and it was fairly high compared to the other methods, whereas the standard deviation had the lowest value (8.5%). For the modified Alsayed method, a 9.2% standard deviation was obtained with a mean error of 3.7%. The results of the analysis proved that the proposed deflection determination method modification described well the deflection phenomenon in the entire load range. However, it should be remembered that the values indicated were slightly underestimated in relation to the test results.

## 6. Conclusions

Based on the studies performed and on the analysis of the results obtained, the following conclusions were formulated:

1. The sand used in the study, being the waste from an all-in aggregate hydroclassification process, was a perfect component in concrete with the addition of steel fibers. This was caused by a high fineness modulus, low changeability factor, and low mineral dust content, as well as the absence of impurities [28].
2. A modification of the sand and concrete mixture with steel fibers and superplasticizer improved the matrix tensile and compressive strength. In the case of preliminarily designed sand concrete of the C12/15 class, the compressive strength of steel fiber fine aggregate concrete equivalent to Classes C25/30 and C30/37 was obtained respectively for fine aggregate concrete with glued fiber bundles 30/0.55 mm and loose fibers 50/0.8.
3. The modified Alsayed method used for deflection calculation proposed in this paper described the relationship between the load and deflection well. When the calculations were compared with the experimental results, an average error of 3.7%, with a standard deviation of 9.2%, was obtained. The EC 2 method of the calculation of plain concrete elements’ deflection indicated, in relation to the analyzed steel fiber fine aggregate concrete beams, a small average error (0.7%) with a higher standard deviation (12.3%). However, it should be remembered that both methods gave values that were slightly underestimated in relation to the experimental values. Therefore, steel fiber fine aggregate concrete element deflection could be safely calculated with the application of the guidelines for plain concrete. Amin, Foster, and Kaufmann’s method gave results lower than the measured ones. The average error was 32.2%. The Bywalski and Kaminski method was the most complicated to use for deflection calculation, but the results were close to the measured values, with an average error of 8%, excluding the D and K series.
4. The plain (commercial) concrete used in this study, with the declared compressive strength Class C35/45, featured higher strength properties compared with the two steel fiber fine aggregate concretes used of lower classes (C25/30 and C30/37). Nevertheless, the deflections of beams (with identical reinforcement ratio) made of steel fiber fine aggregate concrete and plain concrete were comparable. Therefore, the beam elements made of steel fiber sand concrete and plain concrete complied with the requirements put on any plain concrete elements in terms of deflection.

## Figures and Tables

**Table 1:** Steel fiber fine aggregate concrete mixture [24].

Concrete	Components [kg/m^3^]					
	Waste Sand [25]	Cement	Water	Superplasticizer “FM 34”	Steel Fiber [26]	
					30/0.55	50/0.8
SFFARC 1 ^1^	1855	378	140	3.83	34	-
SFFARC 2 ^2^	1835	374	150	3.78	-	33

^1^ SFFARC—Steel fiber fine aggregate concrete with glued fiber bundles; plastic mixture consistence; ^2^ SFFARC—steel fiber fine aggregate concrete with loose fibers; plastic mixture consistence.

**Table 2:** C35/45 commercial (plain) concrete composition for 1 m^3^.

Position	Material	Unit of Measure	Quantity
1	Aggregate (0–2)	kg	646.0
2	Aggregate (2–8)	kg	576.0
3	Aggregate (8–16)	kg	523.0
4	Cement CEM I 32.5 R	kg	410.0
5	Pantarhit® 45 (BV)	liter	1.64
6	Water	liter	188.0

Plastic mixture consistence; fineness modulus 30%.

**Table 3:** The beam and small sized elements’ testing program.

Concrete	Series	Beam Elements				Small Sized Elements
		Beam Marking	Beam No.	Dimensions	Reinforcement (Top/Bottom)	Number of Samples
SFFARC 2	A	A-1, A-2	2	150 mm × 200 mm × 3300 mm	2 # 8/3 # 8	6^1^ + 6^2^ + 3^3^ + 3^4^
	B	B-1, B-2	2		2 # 8/3 # 10	6^1^ + 6^2^ + 3^3^ + 3^4^
	C	C-1, C-2	2		2 # 8/3 # 12	6^1^ + 6^2^ + 3^3^ + 3^4^
	D	D-1, D-2	2		-/3 # 8	6^1^ + 6^2^ + 3^3^ + 3^4^
	G	G-1, G-2	2		2 # 8/3 # 14	6^1^ + 6^2^ + 3^3^ + 3^4^
Plain	F	F-1, F-2	2		2 # 8/3 # 10	12^1^ + 12^2^ + 3^3^ + 3^4^
SFFARC 1	H	H-1, H-2	2		2 # 8/3 # 14	6^1^ + 6^2^ + 3^3^ + 3^4^
	I	I-1, I-2	2		2 # 8/3 # 12	6^1^ + 6^2^ + 3^3^ + 3^4^
	J	J-1, J-2	2		2 # 8/3 # 10	6^1^ + 6^2^ + 3^3^ + 3^4^
	K	K-1, K-2	2		2 # 8/3 # 8	6^1^ + 6^2^ + 3^3^ + 3^4^
		Total	**20 beams**		Total	**192 samples**

^1^ Cubes for determination of compression strength (150 mm × 150 mm × 150 mm); ^2^ cubes for determination of split tensile strength (150 mm × 150 mm × 150 mm); ^3^ cylinders for determination of cylinder compressive strength (150 mm × 300 mm); ^4^ cylinders for determination of the modulus of elasticity (150 mm × 300 mm).

**Table 4:** Ribbed steel mechanical properties obtained in the tests.

Diameter (mm)	Rod Cross-Section (mm^2^)	Yield Point (MPa)	Tensile Strength (MPa)	Modulus of Elasticity (GPa)
8.2	53.0	424	695	223
10.2	81.1	454	714	220
12.2	116.7	430	678	205
14.3	160.6	451	688	214

**Table 5:** Analysis of the deflection of beams in their mid-span at the operational load level.

Beam Series	$\frac{M_{\exp}}{M_{\max}}$	Deflection (mm)						Analysis of Method Used				
		Test Results	Calculation Method Used:					$\frac{a_{\exp.}-a_{method}}{a_{\exp.}}\;(\%)$				
			[22]	[14]	[16]	[17]	Modified Alsayed					
		*a_exp_*	*a_EC2_*	*a_Tan._*	*a_Byw._*	*a_Ami._*	*a_new._*	*a_EC2_*	*a_Tan._*	*a_Byw._*	*a_Ami._*	*a_new._*
A	0.59	8.05	8.52	4.93	7.72	4.42	7.45	−5.8	38.8	4.1	45.1	7.5
B	0.62	10.70	9.89	8.10	9.29	7.11	9.75	7.6	24.3	13.2	33.6	8.9
C	0.58	10.44	9.21	8.62	9.41	7.23	9.21	11.8	17.4	9.8	30.7	11.8
D	0.62	8.74	9.92	6.91	14.87	5.86	8.83	−13.5	20.9	−70.1	33.0	−1.0
F	0.66	10.12	9.65	-	-	-	9.66	4.6	-	-	-	4.5
G	0.58	12.01	11.11	10.82	11.09	8.95	11.17	7.5	9.9	7.7	25.4	7.0
H	0.56	11.83	11.51	8.47	12.00	9.11	11.56	2.7	28.4	−1.5	23.0	2.3
I	0.59	11.81	10.23	9.75	10.16	7.33	10.31	13.4	17.4	10.2	37.9	12.7
J	0.65	11.28	10.82	9.40	10.02	7.32	10.89	4.1	16.7	11.2	35.1	3.5
K	0.60	8.34	10.49	7.05	14.70	6.20	9.98	−25.8	15.5	−76.2	25.6	−19.7
Mean error (%)								0.7	21.0	−10.2	32.2	3.7
Standard deviation (%)								12.3	8.5	36.0	6.9	9.2

$M_{\exp}$, bending moment equivalent to the operational load; $M_{\max}$, bending moment equivalent to the destructive load.
